# Role of *STAT4 *polymorphisms in systemic lupus erythematosus in a Japanese population: a case-control association study of the *STAT1-STAT4 *region

**DOI:** 10.1186/ar2516

**Published:** 2008-09-19

**Authors:** Aya Kawasaki, Ikue Ito, Koki Hikami, Jun Ohashi, Taichi Hayashi, Daisuke Goto, Isao Matsumoto, Satoshi Ito, Akito Tsutsumi, Minori Koga, Tadao Arinami, Robert R Graham, Geoffrey Hom, Yoshinari Takasaki, Hiroshi Hashimoto, Timothy W Behrens, Takayuki Sumida, Naoyuki Tsuchiya

**Affiliations:** 1 Molecular and Genetic Epidemiology Laboratory, Doctoral Program in Life System Medical Sciences, Graduate School of Comprehensive Human Sciences, University of Tsukuba, 1-1-1 Tennodai, Tsukuba 305-8575, Japan; 2 Division of Clinical Immunology, Doctoral Program in Clinical Sciences, Graduate School of Comprehensive Human Science, University of Tsukuba, 1-1-1 Tennodai, Tsukuba 305-8575, Japan; 3 Department of Medicine, Takikawa Municipal Hospital, 2-2-34 Omachi, Takikawa 073-0033, Japan; 4 Department of Medical Genetics, Doctoral Program in Life System Medical Sciences, Graduate School of Comprehensive Human Sciences, University of Tsukuba, 1-1-1 Tennodai, Tsukuba 305-8575, Japan; 5 Genentech, Inc., 1 DNA Way, South San Francisco, CA 94080, USA; 6 Division of Rheumatology, Department of Internal Medicine, 2-1-1 Hongo, Bunkyo-ku, Tokyo 113-8421, Japan

## Abstract

**Introduction:**

Recent studies identified *STAT4 *(signal transducers and activators of transcription-4) as a susceptibility gene for systemic lupus erythematosus (SLE). *STAT1 *is encoded adjacently to *STAT4 *on 2q32.2-q32.3, upregulated in peripheral blood mononuclear cells from SLE patients, and functionally relevant to SLE. This study was conducted to test whether *STAT4 *is associated with SLE in a Japanese population also, to identify the risk haplotype, and to examine the potential genetic contribution of *STAT1*. To accomplish these aims, we carried out a comprehensive association analysis of 52 tag single nucleotide polymorphisms (SNPs) encompassing the *STAT1*-*STAT4 *region.

**Methods:**

In the first screening, 52 tag SNPs were selected based on HapMap Phase II JPT (Japanese in Tokyo, Japan) data, and case-control association analysis was carried out on 105 Japanese female patients with SLE and 102 female controls. For associated SNPs, additional cases and controls were genotyped and association was analyzed using 308 SLE patients and 306 controls. Estimation of haplotype frequencies and an association study using the permutation test were performed with Haploview version 4.0 software. Population attributable risk percentage was estimated to compare the epidemiological significance of the risk genotype among populations.

**Results:**

In the first screening, rs7574865, rs11889341, and rs10168266 in *STAT4 *were most significantly associated (*P *< 0.01). Significant association was not observed for *STAT1*. Subsequent association studies of the three SNPs using 308 SLE patients and 306 controls confirmed a strong association of the rs7574865T allele (SLE patients: 46.3%, controls: 33.5%, *P *= 4.9 × 10^-6^, odds ratio 1.71) as well as TTT haplotype (rs10168266/rs11889341/rs7574865) (*P *= 1.5 × 10^-6^). The association was stronger in subgroups of SLE with nephritis and anti-double-stranded DNA antibodies. Population attributable risk percentage was estimated to be higher in the Japanese population (40.2%) than in Americans of European descent (19.5%).

**Conclusions:**

The same *STAT4 *risk allele is associated with SLE in Caucasian and Japanese populations. Evidence for a role of *STAT1 *in genetic susceptibility to SLE was not detected. The contribution of *STAT4 *for the genetic background of SLE may be greater in the Japanese population than in Americans of European descent.

## Introduction

Systemic lupus erythematosus (SLE) is a complex disease characterized by autoantibody production and involvement of multiple organs, including kidneys. Both genetic and environmental factors contribute to the development of SLE [1]. Until now, several genes have been reported to be associated with SLE, of which interferon regulatory factor-5 (*IRF5*) has been identified as a susceptibility gene common to multiple populations [2-6]. Recently, association of *STAT4 *(signal transducers and activators of transcription-4) haplotype tagged by rs7574865T with SLE was demonstrated in Caucasians [7]. Subsequently, two genome-wide association studies [8,9], a study focused on the *STAT4 *region in Caucasians [10], and replication studies in Colombians [11] and a Japanese population [12] have confirmed the association. In addition, an association of *STAT4 *with SLE phenotypes such as anti-double-stranded DNA (anti-dsDNA) autoantibodies, renal disorder, and age at diagnosis was reported [10,13]. An association of rs7574865 with other autoimmune diseases such as rheumatoid arthritis and primary Sjögren syndrome has also been demonstrated [7,11,12,14]. The *STAT4 *gene encodes a transcription factor belonging to the STAT family expressed in lymphocytes, macrophages, and dendritic cells. STAT4 is essential for interleukin (IL)-12 signaling and induces interferon-gamma (IFNγ) production and Th1 differentiation [15]. STAT4 is also activated by type I IFNs (IFNα/β) [16]. Moreover, the requirement of STAT4 in IL-23-induced IL-17 production has been suggested [17]. Two isoforms of STAT4, STAT4α and STAT4β, are known [18]. Expression of STAT4β, lacking the transactivation domain, did not appear to be affected by the *STAT4 *single nucleotide polymorphisms (SNPs) [13]. STAT1, another member of the STAT family, is activated by type I IFNs and IFNγ and plays an important role in immune responses [19]. STAT1 has been reported to be upregulated in peripheral blood mononuclear cells from SLE patients and in kidneys of lupus mice with nephritis [20,21], suggesting that STAT1 may play a role in the pathogenesis of SLE. A possible role of SNPs in the *STAT1-STAT4 *region other than the haplotype tagged by rs7574865T has recently been excluded in Caucasians [10]. However, in view of substantial differences in disease-associated alleles among populations [2], such analysis should be performed in each population. In this study, we carried out a comprehensive association analysis of the *STAT1*-*STAT4 *region with SLE in a Japanese population by scanning 52 tag SNPs of the region encompassing *STAT1 *and *STAT4*.

## Materials and methods

### Patients and healthy controls

Patients and controls were recruited at Juntendo University, the University of Tsukuba, and the University of Tokyo. All patients and healthy controls were unrelated Japanese persons living in the central part of Japan. Three hundred eight SLE patients (18 males and 290 females, average age 41.4 ± 13.5 years) and 306 healthy individuals (119 males and 187 females, average age 32.6 ± 9.8 years) were studied. Diagnosis of SLE and classification of the patients into clinical subsets were carried out according to the American College of Rheumatology criteria for SLE [22]. There was no overlap in cases or controls between this study and the recently reported study in a Japanese population [12]. These studies were reviewed and approved by the research ethics committees of the University of Tsukuba, the University of Tokyo, and Juntendo University. Informed consent was obtained from all study participants.

### Association study

Fifty-two tag SNPs in the *STAT1*-*STAT4 *region were selected with an *r*^2 ^threshold of 0.9 based on the HapMap Phase II JPT (Japanese in Tokyo, Japan) data. These tag SNPs captured 127 SNPs with a minor allele frequency of greater than or equal to 0.05. First screening was performed in 105 Japanese female SLE patients and 102 female healthy controls using the GoldenGate SNP genotyping assay (Illumina, Inc., San Diego, CA, USA). For the three SNPs that exhibited significant association (*P *< 0.01), additional samples were genotyped using the TaqMan SNP Genotyping Assay (Applied Biosystems, Foster City, CA, USA), and association was examined in 308 SLE patients and 306 healthy individuals.

### Statistical analysis

Association of each SNP was analyzed by chi-square test. Because of the replicative nature of this study, correction for multiple testing was not performed, and unadjusted *P *values are shown. Haplotype frequency estimation and association analysis using the permutation test were performed with Haploview version 4.0 software (Broad Institute of MIT and Harvard, Cambridge, MA, USA). In the haplotype analysis, the genotype data for rs10168266, rs11889341, and rs7574865 were used and these SNPs were assumed to compose a single haplotype block. In the permutation test, only frequencies of haplotypes in this block were compared (that is, the 'Haplotypes in Blocks Only' option was used). Ten million permutations were performed. To test the significance of each SNP conditional on the genotypes of other SNPs, logistic regression analysis was performed under the additive model for the minor allele. Assuming a polymorphic site with two alleles A and a, genotypes were encoded as 0 = aa, 1 = Aa, and 2 = AA. Population attributable risk percentage (PAR%) for the risk genotype (rs7574865T/T and T/G) was estimated by the formula

PAR% = Pe (RR - 1)/(Pe [RR - 1] + 1),

where Pe represents the risk genotype frequency in the population and RR represents relative risk of the risk genotype [23]. Given the low prevalence of SLE, Pe can be estimated based on the genotype frequencies in healthy controls and RR can be approximated by odds ratio (OR) for the risk genotypes.

## Results and Discussion

**Figure 1:**
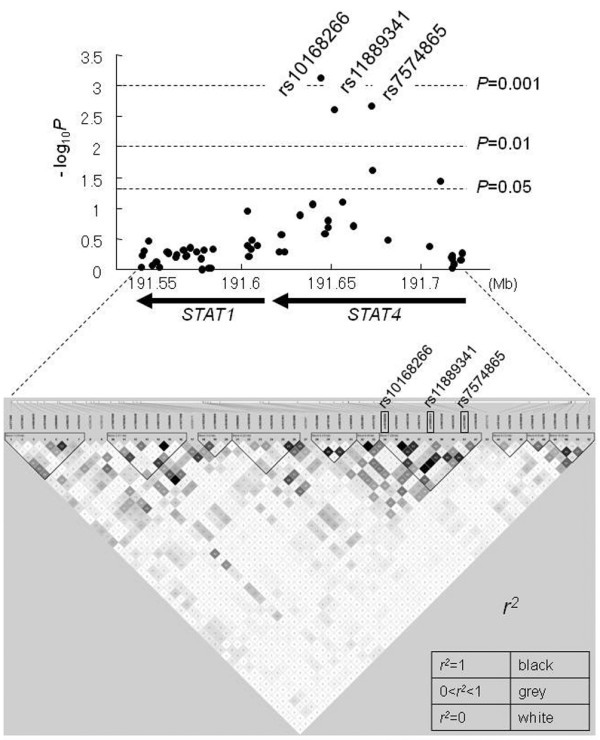
Linkage disequilibrium plot of the *STAT1-STAT4 *region in a Japanese population and first screening of 52 tag single nucleotide polymorphisms (SNPs). In the upper panel, *P *values for differences in allele frequencies were calculated by chi-square test using two-by-two contingency tables. The -log *P *value for each SNP is shown. In the lower panel, *r*^2 ^values calculated using Haploview version 4.0 software based on data from 102 healthy individuals are shown. The location and direction of transcription of *STAT1 *and *STAT4 *are indicated by arrows. SNPs rs10168266, rs11889341, and rs7574865 belong to the same haplotype block.

The *STAT4 *gene is located on 2q32.2-q32.3 adjacently to *STAT1 *gene, and the region encompassing *STAT1 *and *STAT4 *spans approximately 180 kilobase pairs. In the first screening, 52 tag SNPs in the *STAT1*-*STAT4 *region, selected with an *r*^2 ^threshold of 0.9 based on the HapMap Phase II JPT data, were genotyped in 105 Japanese female SLE patients and 102 female healthy controls, and allele frequencies were compared between SLE patients and controls. A linkage disequilibrium (LD) plot and the results of the association study in the *STAT1*-*STAT4 *region are shown in Figure 1. Pairwise *r*^2 ^values between 52 tag SNPs were calculated using genotyping data from 102 healthy individuals.

Among the tag SNPs, rs10168266C>T, rs11889341C>T, and rs7574865G>T were most significantly associated with SLE in the first screening (*P *< 0.01). Allele frequencies of rs10168266T, rs11889341T, and rs7574865T were significantly increased in SLE compared with healthy controls (Table 1 and Figure 1). These SNPs were located in the introns of *STAT4 *and in LD with each other. In contrast, significant association was not detected for SNPs in the *STAT1 *region (*P *> 0.05).

**Table 1:** Minor allele frequencies and *P *values for 52 tag single nucleotide polymorphisms in the *STAT1*-*STAT4 *region in the first screening

			Minor allele frequency		
SNP	Chromosomal position^a^	Minor allele	SLE patients (n = 105)	Controls (n = 102)	*P *value
rs3771300	191543841	C	0.305	0.309	0.929
rs7575823	191544163	A	0.167	0.147	0.584
rs16824035	191545879	A	0.057	0.074	0.500
rs1914408	191548221	A	0.271	0.314	0.344
rs2066804	191550004	A	0.471	0.480	0.855
rs2280235	191552075	A	0.486	0.471	0.758
rs3755312	191554236	C	0.181	0.176	0.905
rs2280234	191558344	G	0.162	0.186	0.513
rs2280232	191559011	C	0.143	0.123	0.543
rs11887698	191563119	G	0.327	0.304	0.629
rs7562024	191563766	G	0.090	0.108	0.554
rs11904548	191567235	A	0.162	0.137	0.482
rs12693591	191568747	A	0.257	0.235	0.606
rs16833155	191569622	A	0.043	0.054	0.600
rs2066805	191571146	G	0.038	0.054	0.442
rs11677408	191574860	A	0.129	0.108	0.514
rs2030171	191577408	G	0.329	0.309	0.666
rs11693463	191578156	G	0.195	0.196	0.983
rs11885069	191578869	A	0.162	0.137	0.482
rs10199181	191581798	T	0.267	0.265	0.964
rs2066802	191582912	G	0.257	0.255	0.956
rs13029532	191584146	C	0.082	0.103	0.457
rs3024904	191603447	A	0.112	0.141	0.400
rs3024936	191603621	C	0.024	0.055	0.112
rs1517351	191604290	C	0.490	0.464	0.602
rs3024896	191604961	A	0.448	0.412	0.461
rs925847	191605785	A	0.538	0.490	0.330
rs3024886	191608694	A	0.457	0.417	0.407
rs6715106	191621279	G	0.067	0.083	0.520
rs16833215	191622044	G	0.495	0.441	0.270
rs1400654	191623918	T	0.066	0.083	0.524
rs3024861	191632851	T	0.471	0.397	0.127
rs1517352	191639709	A	0.481	0.397	0.086
rs10168266	191644049	A	0.400	0.245	7.6 × 10^-4^
rs7594501	191646845	A	0.114	0.152	0.250
rs16833239	191648505	A	0.110	0.152	0.200
rs7601754	191648696	G	0.129	0.178	0.162
rs11889341	191651987	A	0.443	0.299	0.003
rs16833249	191656517	G	0.567	0.480	0.079
rs6434435	191662109	A	0.099	0.141	0.192
rs7574865	191672878	A	0.471	0.324	0.002
rs12463658	191673589	C	0.581	0.471	0.025
rs6752770	191681808	G	0.205	0.245	0.326
rs1551443	191704763	A	0.238	0.206	0.431
rs2356350	191710783	G	0.510	0.407	0.036
rs10189819	191716994	G	0.133	0.118	0.630
rs7596818	191717555	A	0.320	0.295	0.580
rs11685878	191717700	A	0.429	0.431	0.954
rs12991409	191717762	G	0.100	0.113	0.674
rs12327969	191719016	G	0.390	0.402	0.811
rs12988825	191722509	C	0.119	0.132	0.683
rs7572482	191723317	G	0.490	0.461	0.545

^a^Chromosomal positions are shown according to the National Center for Biotechnology Information (Bethesda, MD, USA) reference assembly. SLE, systemic lupus erythematosus; SNP, single nucleotide polymorphism; STAT, signal transducers and activators of transcription.

To confirm the association detected in the first screening, additional patients and controls were genotyped for the three SNPs using the TaqMan SNP Genotyping Assay, and association was examined in 308 SLE patients and 306 healthy controls in total (Table 2). Significant deviation from Hardy-Weinberg equilibrium was not detected in healthy controls (*P *> 0.05). The rs7574865T allele, previously shown to be associated with SLE in Caucasians, was significantly increased in SLE patients (46.3%) compared with controls (33.5%, *P *= 4.9 × 10^-6^, OR 1.71). The association was compatible with the dominant model, under which the OR was 2.19 (T/T + G/T versus G/G).

**Table 2:** Association of *STAT4 *single nucleotide polymorphisms rs10168266, rs11889341, and rs7574865 with systemic lupus erythematosus

	SLE patients (n = 308)		Healthy controls (n = 306)		*P *value	Odds ratio	95% CI
			
	Number	Percentage	Number	Percentage			
rs10168266							
Genotype frequency							
C/C	118	38.3	166	54.2			
C/T	147	47.7	122	39.9	7.5 × 10^-5a^	1.91	1.39–2.63^a^
T/T	43	14.0	18	5.9			
Allele frequency							
T	233	37.8	158	25.8	6.3 × 10^-6^	1.75	1.37–2.23
rs11889341							
Genotype frequency							
C/C	99	32.1	153	50.0			
C/T	161	52.3	126	41.2	6.9 × 10^-6a^	2.11	1.52–2.92^a^
T/T	48	15.6	27	8.8			
Allele frequency							
T	257	41.7	180	29.4	6.6 × 10^-6^	1.72	1.36–2.17
rs7574865							
Genotype frequency							
G/G	80	26.0	133	43.5			
G/T	171	55.5	141	46.1	5.3 × 10^-6a^	2.19	1.56–3.07^a^
T/T	57	18.5	32	10.5			
Allele frequency							
T	285	46.3	205	33.5	4.9 × 10^-6^	1.71	1.36–2.15
rs10168266/rs11889341/rs7574865							
Haplotype frequency							
CCG		52.7		65.0	1.0 × 10^-5b^		
TTT		36.8		24.3	1.5 × 10^-6b^		
CCT		4.9		5.1	NS^b^		
CTT		4.6		4.1	NS^b^		

^a^*P *values, odds ratios, and 95% confidence intervals (CIs) were calculated under the dominant model for the minor allele. ^b^*P *values were calculated by permutation test using Haploview version 4.0 software. Ten million permutations were performed. NS, not significant; SLE, systemic lupus erythematosus; STAT, signal transducers and activators of transcription.

The SNPs rs11889341 and rs10168266 were in LD with rs7574865 (*r*^2^: 0.57 to 0.78, *D'*: 0.91 to 0.97) and were also significantly associated with SLE (allele frequency: *P *= 6.6 × 10^-6 ^and *P *= 6.3 × 10^-6^, respectively). Haplotype analysis revealed that the haplotype carrying rs10168266T, rs11889341T, and rs7574865T was significantly increased (SLE: 36.8%, control: 24.3%, *P *= 1.5 × 10^-6^) whereas the haplotype carrying 10168266C, rs11889341C, and rs7574865G was significantly decreased in SLE (SLE: 52.7%, control: 65.0%, *P *= 1.0 × 10^-5^). Logistic regression analysis demonstrated that the association of each SNP lost statistical significance when adjusted for genotype of the other SNPs (Table 3). Thus, due to the strong LD, it was impossible to identify a single causative SNP among the three.

**Table 3:** Logistic regression analysis of the systemic lupus erythematosus-associated single nucleotide polymorphisms in *STAT4*

		*P *adjusted for		
SNP	*P *value	rs10168266	rs11889341	rs7574865

rs10168266	4.9 × 10^-6^	NA	0.272	0.146
rs11889341	4.7 × 10^-6^	0.251	NA	0.388
rs7574865	2.1 × 10^-6^	0.052	0.130	NA

NA, not applicable; SNP, single nucleotide polymorphism; STAT, signal transducers and activators of transcription.

We next tested whether *STAT4 *rs7574865 was associated with phenotypes of SLE such as presence of nephritis, anti-dsDNA antibodies, and early age of onset (less than 20 years) as *STAT4 *genotype has been shown to be more strongly associated with subgroups of SLE with these phenotypes [10] (Table 4). Association of rs7574865 was observed both in SLE patients with nephritis (*P *= 1.0 × 10^-5^, OR = 1.85) and in those without nephritis (*P *= 0.0031, OR = 1.55). The association was stronger in SLE patients with nephritis, although the difference between SLE with and without nephritis (case-only analysis) did not reach statistical significance. Similarly, rs7574865T was significantly increased in SLE patients with anti-dsDNA antibodies compared with healthy controls, whereas association was not detected in SLE patients without anti-dsDNA antibodies. The frequency of rs7574865T was slightly higher in the patients with an age of onset of less than 20 years as compared with greater than or equal to 20 years, although the difference was not statistically significant. These tendencies are consistent with those reported in Caucasians [10]. These interpretations were not affected when the significance level was corrected for the number of comparisons (three phenotypes).

**Table 4:** Association of *STAT4 *rs7574865 with characteristics of systemic lupus erythematosus such as nephritis, age of onset, and anti-double-stranded-DNA antibodies

	T allele		*P *value	Odds ratio (95% CI)
	Number	Frequency		

Case subgroup versus healthy controls				
Nephritis				
Present (n = 165)	159	48.2%	1.0 × 10^-5^	1.85 (1.41–2.42)
Absent (n = 138)	121	43.8%	0.0031	1.55 (1.16–2.07)
Anti-double-stranded DNA antibodies				
Present (n = 130)	125	48.1%	4.9 × 10^-5^	1.84 (1.37–2.47)
Absent (n = 34)	24	35.3%	NS	1.08 (0.64–1.83)
Age of onset				
<20 years (n = 86)	83	48.3%	3.9 × 10^-4^	1.85 (1.32–2.60)
≥20 years (n = 198)	180	45.5%	1.4 × 10^-4^	1.65 (1.28–2.14)
Healthy controls (n = 306)	205	33.5%		
Case-only (present versus absent or <20 versus ≥ 20 years)				
Nephritis			NS	1.19 (0.86–1.64)
Anti-double-stranded DNA antibodies			NS	1.70 (0.98–2.95)
Age of onset			NS	1.12 (0.78–1.60)

Systemic lupus erythematosus (SLE) patients were stratified into subgroups according to the presence or absence of nephritis, anti-double-stranded DNA (anti-dsDNA) antibodies, and age of onset (<20 or ≥ 20 years). Allele frequencies were compared between each SLE subgroup and healthy controls as well as between SLE subgroups (case-only analysis, nephritis present versus absent, anti-dsDNA antibodies present versus absent, and age of onset <20 versus ≥ 20 years). CI, confidence interval; NS, not significant; STAT, signal transducers and activators of transcription.

To evaluate the epidemiological significance of *STAT4 *polymorphism in the genetic background of SLE in the Japanese population, we estimated the PAR% in Japanese persons and Caucasians using our present data and previously reported data [8,11,12] (Table 5). Because the frequency and OR of the risk genotype of rs7574865 were greater in the Japanese population than those of North Americans of European descent [8], PAR% in the Japanese population (40.2%) was much higher than that of the latter (19.5%). A similarly high PAR% was observed in two of the three Japanese case-control series reported by Kobayashi and colleagues [12] and in Colombians [11]. Because PAR% may be affected by the difference in the method of ascertainment of each study, this comparison may not be completely valid. Nevertheless, these observations suggested that the contribution of *STAT4 *for SLE is greater in the Japanese population as compared with the Americans of European descent.

**Table 5:** Population attributable risk percentage of *STAT4 *rs7574865 under the dominant model

Population [reference]	Frequency of (T/T+T/G)	Odds ratio	PAR%
Japanese (this study)	56.5%	2.19	40.2%
Japanese (TWMU) [12]	52.3%	1.81	29.7%
Japanese (RIKEN) [12]	51.7%	1.51	20.8%
Japanese (Tokushima/Fukuoka) [12]	51.9%	2.07	35.8%
Americans of European descent [8]	41.2%	1.59	19.5%
Colombians [11]	51.7%	1.87	31.0%

PAR%, population attributable risk percentage; RIKEN, The Institute of Physical and Chemical Research, Wako, Japan; STAT, signal transducers and activators of transcription; TWMU, Tokyo Women's Medical University, Tokyo, Japan.

At this point, molecular mechanisms that account for the association of *STAT4 *intron SNPs with SLE remain unclear. Studies with lupus model mice lacking *Stat4 *showed conflicting results. *Stat4 *deficiency reduced nephritis and autoantibody production in B6.NZM.*Sle1.Sle2.Sle3 *mice [24]. In contrast, *Stat4*-deficient NZM (New Zealand mixed) mice developed accelerated nephritis and increased mortality in the absence of high levels of autoantibodies [25]. STAT4 has been shown to be involved in the induction of IFNγ, differentiation of Th1 and Th17 cells, and signal transduction from type I IFN receptors [15]. Th1 cytokines, especially IFNγ, have been shown to play a role in the pathogenesis of lupus nephritis [26]. Recently, T cells from SLE patients were shown to produce excessive amounts of IFNγ upon stimulation [27]. These observations may implicate the role of *STAT4 *SNPs in IFNγ production.

The role of type I IFNs in SLE has been established [1]. Elevated serum type I IFN levels and expression of IFN-inducible genes in peripheral mononuclear cells were reported in SLE [28,29]. The association of *IRF5*, which induces type I IFNs, with SLE has been established [2-6]. STAT4 is activated by type I IFN as well as IL-12 signals and produces IFNγ [15]. Thus, STAT4 may also contribute to SLE as a component of the type I IFN signal pathway. Furthermore, STAT4 has been reported to transduce IL-12 signals to induce IFNγ production in B cells [30].

It is interesting to note that significant association of *STAT4 *was not observed in SLE patients without anti-dsDNA antibodies (Table 4). It would have been interesting to examine the effect of the genotype on the levels, rather than presence or absence, of anti-dsDNA antibody However, because the antibody levels fluctuate in association with disease activity and treatment, association with the genotype should be examined using the lifetime highest anti-dsDNA antibody level of each patient. Such data were not available for this study, and we hope that we can address this issue in the future.

Most of these observations imply that *STAT4 *risk genotype may be associated with an elevated expression level and/or function of STAT4 protein. A recent study reported that the *STAT4 *risk allele was associated with overexpression of *STAT4 *in osteoblasts but not in B cells [13]. To address the significance of such findings, it will be necessary to examine the effect of this genotype on the expression levels and splicing isoforms in T and B cells.

## Conclusion

Through comprehensive association analysis of the *STAT1*-*STAT4 *region with SLE in the Japanese population, we demonstrated that the same *STAT4 *risk allele in Caucasians was strongly associated with susceptibility to SLE in the Japanese population. In contrast, evidence for an association of *STAT1 *SNPs was not observed. The contribution of *STAT4 *SNPs to the genetic background of SLE may be greater in the Japanese population than in Americans of European descent.

## Abbreviations

anti-dsDNA: anti-double-stranded DNA; CI: confidence interval; IFN: interferon; IL: interleukin; IRF5: interferon regulatory factor-5; JPT: Japanese in Tokyo, Japan; LD: linkage disequilibrium; OR: odds ratio; PAR%: population attributable risk percentage; RR: relative risk; SLE: systemic lupus erythematosus; SNP: single nucleotide polymorphism; STAT: signal transducers and activators of transcription.

## Competing interests

RRG, GH, and TWB are employees of and hold stocks or shares in Genentech, Inc. (South San Francisco, CA, USA). The other authors declare that they have no competing interests.

## Authors' contributions

AK participated in the study design, carried out all genotyping and statistical analyses, and wrote the manuscript. II, KH, MK, and TA participated in the first screening using Illumina GoldenGate assay (with AK), including tag SNP selection, genotyping, and statistical analysis. JO carried out statistical analysis with AK and helped in the manuscript preparation. TH, DG, IM, SI, AT, YT, HH, and TS recruited Japanese patients with SLE and collected clinical information. RRG and GH provided Caucasian data. NT conceived of the study, together with TWB, and participated in its design and coordination, recruited patients and controls, and helped in the manuscript preparation. All authors read and approved the final manuscript.
